# Cognitive Impairments in Viral Hepatitis Patients: Causes, Manifestations, and Impact on Quality of Life

**DOI:** 10.5041/RMMJ.10539

**Published:** 2025-01-30

**Authors:** Tatyana Vasiliyevna Polukchi

**Affiliations:** Department of Neurosurgery, South Kazakhstan Medical Academy, Shymkent, Republic of Kazakhstan

**Keywords:** Cognitive impairment, hepatic encephalopathy, neurocognitive function, quality of life, viral hepatitis

## Abstract

Viral hepatitis, primarily caused by hepatitis B virus and hepatitis C virus, is widely recognized for its impact on liver function, but emerging evidence suggests it also affects cognitive function. This review explores the causes, manifestations, and impact of cognitive impairments in patients with viral hepatitis, to better understand this often-overlooked aspect of the disease. A literature review was conducted, focusing on studies published in PubMed up to August 2024. Key areas covered include the pathophysiological mechanisms behind cognitive impairment in viral hepatitis, clinical manifestations observed in affected patients, the implications for their daily functioning and overall well-being, and the tools used in cognitive assessments. Common manifestations included deficits in attention, memory, executive function, and psychomotor speed. These cognitive challenges can significantly impact daily activities, occupational performance, and social interactions, contributing to reduced quality of life. Cognitive impairments in viral hepatitis patients represent a significant concern that extends beyond liver health. Recognizing and addressing these cognitive issues are crucial for improving patient outcomes. Enhanced diagnostic strategies and targeted interventions are needed to better manage cognitive symptoms and support affected individuals in maintaining their quality of life. This narrative review aims to enhance clinical practice and inform future research directions.

## INTRODUCTION

Viral hepatitis encompasses a range of liver infections caused by distinct viruses, each with unique epidemiological and clinical profiles. These infections lead to inflammation of the liver and can vary in severity from asymptomatic to life-threatening conditions.^1^ Five major, biologically distinct hepatotropic viruses (A, B, C, D, and E) account for most of the global burden of viral hepatitis. Hepatitis A and hepatitis E are primarily transmitted through the fecal–oral route.^2^ Hepatitis B and hepatitis C are primarily transmitted through blood and bodily fluids and are notably associated with a high number of chronic infections.^2^,^3^ The disease can lead to chronic liver conditions, including cirrhosis and hepatocellular carcinoma, and significantly affects quality of life and overall health.^4^,^5^ Both hepatitis B and C virus (HBV and HCV) represent a significant health concern, with 254 million and 50 million infected individuals worldwide, respectively.^4^,^6^ In 2022, approximately 1.2 million new HBV and nearly 1 million new HCV infections were reported.^6^–^8^ Despite notable progress in prevention and control of viral hepatitis, significant challenges remain, particularly in regions where the disease is endemic.^2^ Chronic viral hepatitis can also have a significant impact on a person’s overall health and well-being.^9^,^10^ Chronic hepatitis, especially chronic hepatitis C, has been associated with cognitive issues such as difficulties with memory, concentration, and mental clarity.^9^,^10^

This narrative review examines the current understanding of the significance, causes, clinical manifestations, diagnosis, and impact of cognitive impairment in patients with viral hepatitis. In so doing, it raises awareness of the need for a multidisciplinary and focused approach toward viral hepatitis that recognizes cognitive impairment as a critical component of patient care.

## SIGNIFICANCE OF COGNITIVE IMPAIRMENT IN PATIENTS WITH VIRAL HEPATITIS

Many individuals with chronic viral hepatitis experience persistent fatigue that is not necessarily proportional to the severity of their disease.^9^,^10^ Recent data indicate that a significant proportion of people with chronic hepatitis C may exhibit both clinical and subclinical manifestations of cognitive disorders. Studies suggest that around 50% of chronic hepatitis C individuals may show some form of cognitive impairment.^11^ Research indicates that subclinical cognitive impairments, such as difficulties with attention, memory, and executive function, are prevalent in chronic viral hepatitis patients.^12^,^13^

More severe cognitive impairments that affect daily functioning have also been reported. Thought processing speed, memory, and executive function have all been documented in chronic viral hepatitis patients.^14^ Hepatitis C virus may exert direct neurotoxic effects, though the exact mechanisms are unclear. Studies suggest that HCV may alter brain function through inflammatory pathways or neurotoxic metabolites.^15^ One prospective interventional study found that hepatitis C patients with high viral loads experienced greater cognitive impairment.^10^ It also demonstrated that HCV neurotoxicity could be partially reversed and that there was subsequent improvement in patient-reported outcomes following treatment. Another study indicated individuals aged 60 years or older with chronic hepatitis C may be at an increased risk of cognitive impairment.^16^

## CAUSES OF COGNITIVE IMPAIRMENT IN VIRAL HEPATITIS

Hepatitis C virus has been more extensively studied in relation to its impact on brain function compared to HBV. Cognitive impairments associated with chronic hepatitis C are well-documented; it may exert direct neurotoxic effects on the brain, though the precise mechanisms remain under investigation.^15^ Chronic hepatitis C leads to systemic inflammation, which is believed to contribute to cognitive impairments. Elevated levels of inflammatory cytokines and other markers of inflammation have been linked to cognitive decline.^17^ Although typically associated with advanced liver disease, even mild hepatic encephalopathy can affect cognitive function in hepatitis C patients.^18^ Neurocognitive impairments in patients with viral hepatitis are likely associated with systemic inflammation, with attention being the most vulnerable area of neurocognitive function. Moreover, there is a suggestion that systemic inflammation caused by HCV may play a role in the development of neurocognitive disorders, the focus of ongoing research.^19^ The decline in neurocognitive functions observed in patients with chronic hepatitis B and hepatitis C, along with the mediating role of systemic inflammation shown in some studies, suggests that this process is gradual. Initially, the increasing activity of chronic viral infection causes systemic stress, which ultimately leads to neurodegeneration.^19^

It is well known that regulatory T cells play a key role in viral hepatitis development by maintaining the balance between immune responses and viral replication. We hypothesize that genetic polymorphisms in genes associated with regulatory T cells, such as interleukin-2, transforming growth factor-β1, forkhead box P3, and adenylate cyclase 9, may influence host immune regulation in the context of viral hepatitis.^20^,^21^ Both hepatitis C and alcohol use disorder negatively affect the immune system, leading to changes in signaling cells and inflammatory processes. Studies have shown that hepatitis C with alcohol use disorder is associated with more pronounced psychological disorders, which may be caused by an enhanced buffering effect, cytokine expression disruption, and blood–brain barrier dysfunction.^22^ Damage to the blood–brain barrier caused by alcoholism may increase the likelihood of neuropathological conditions in the context of chronic diseases.

Advanced HBV-related liver disease can lead to hepatic encephalopathy, which significantly impacts cognitive function. Even subclinical hepatic encephalopathy can contribute to cognitive issues.^23^ The potential for HBV to have direct neurotoxic effects on the brain is still under investigation, but systemic inflammation and liver dysfunction are known to affect brain function.^24^

The development of various antiviral agents has significantly advanced the treatment of chronic viral hepatitis, particularly hepatitis B and hepatitis C. While these therapies are effective in managing viral infections and improving liver health, they are not without potential side effects, including neurotoxicity. Interferon-alpha, once a cornerstone of hepatitis C treatment, is known for its neuropsychiatric side effects, including depression, anxiety, and cognitive impairment. These effects are attributed to the drug’s impact on the central nervous system and the induction of systemic inflammation.^25^ Direct-acting antivirals such as sofosbuvir, ledipasvir, and daclatasvir have a more favorable safety profile compared to interferon. However, rare cases of neuropsychiatric symptoms, including mood disorders and cognitive issues, have been reported.^26^ Nucleos(t)ide analogs such as tenofovir, entecavir, and lamivudine are commonly used in hepatitis B treatment and are generally considered to have a low neurotoxicity risk. However, tenofovir has been associated with renal and bone toxicity, which may indirectly affect neurological function.^27^

Some studies suggest that cognitive impairments associated with HCV infection may partially regress after successful treatment. For example, patients with hepatitis C who underwent treatment for 12 weeks showed partially improved cognitive functions.^10^ However, another treatment achieved favorable results in a broader group of hepatitis C patients, including those with cirrhosis. Significant improvements in symptoms and disease regression were observed after using direct-acting antiviral drugs for chronic hepatitis C treatment. It is worth noting that Chemello et al. focused particularly on neurological disorders and concluded that, in some cases, there was an improvement in neurological status, especially after achieving viral eradication.^26^ Other studies have shown a significant positive correlation between coffee consumption (three or more cups per day) and improvements in verbal speed, psychomotor speed (coding), and executive functions. Thus, regular coffee consumption may help preserve neurocognitive functions.^28^

## CLINICAL MANIFESTATIONS OF COGNITIVE IMPAIRMENT

Cognitive impairment in the context of viral hepatitis presents in various ways and may significantly affect quality of life. Cognitive impairment in hepatitis B patients is less well-documented compared to hepatitis C. However, studies have indicated that chronic hepatitis B may contribute to neurocognitive disturbances.^9^ In contrast, the relationship between hepatitis C and cognitive impairment has been more extensively studied. Many patients with chronic hepatitis C report difficulties with attention and concentration, which may manifest as trouble focusing on tasks, frequent distractibility, and difficulties maintaining mental clarity.^13^ Impairments in short-term memory and working memory are commonly observed. Patients may struggle to recall recent events or follow conversations, which can impact daily functioning.^29^ Problems with executive functions, such as planning, organizing, and decision-making, have also been noted, which can lead to difficulties managing personal affairs or adhering to treatment regimens.^9^,^30^ Reduced psychomotor speed has been reported often; patients experienced slower reaction times and diminished processing speed.^9^,^12^,^31^

Tan et al. recently showed that chronic hepatitis B patients exhibited lower verbal comprehension scores and information and matrix reasoning indices compared to the control group, as well as a decline in language functions compared to hepatitis C patients.^19^ In addition to the negative impact of chronic hepatitis B on language and executive functions, Tan et al. also revealed that patients with chronic HCV infection performed worse on tasks related to executive functions, psychomotor speed, memory, and attention, while visual-spatial and language functions remained unchanged. Patients with chronic hepatitis C exhibited the lowest performance on tasks related to executive functions, psychomotor speed, memory, and attention, compared to patients with chronic hepatitis B. Different deficit profiles identified in neuropsychological tests may also indicate various characteristics in the mechanisms leading to neurocognitive impairments in patients with chronic hepatitis. They concluded that the deficits identified in patients with chronic hepatitis B in the area of language functions may be associated with cortical dementia syndromes, while the decline in attention and memory in patients with chronic hepatitis C was more closely related to subcortical dementia syndrome.^19^

## DIAGNOSIS OF COGNITIVE IMPAIRMENT

Cognitive impairment encompasses a range of difficulties in mental processes such as memory, attention, and executive function. Accurate diagnosis is critical for effective management and treatment. Clinical evaluation is the cornerstone of diagnosing cognitive impairment. This involves a detailed patient history, clinical interviews, and behavioral observations.^9^ Table 1 provides a summary of the plethora of tools used to diagnose cognitive impairment.

**Table 1:** Overview of Tools Used in Diagnosing and Treating Cognitive Impairment.

Tool	Purpose
**Neuropsychological Test Tools**	
Mini-Mental State Examination (MMSE)^32^	Used for initial cognitive screening, assessing orientation, memory, attention, and language
Montreal Cognitive Assessment (MoCA)^13^,^33^	Designed for detecting mild cognitive impairment (MCI), assessing a broad range of cognitive functions
MoCA-CFS Composite Score^34^	Used to evaluate hepatic encephalopathy severity and predict health deterioration
Wechsler Adult Intelligence Scale (WAIS)^35^	Provides insights into various cognitive functions like memory, executive function, and processing speed
Cambridge Neuropsychological Test Automated Battery (CANTAB)^36^	Provides detailed insights into cognitive functions such as memory, executive function, and processing speed
Neuropsychological Assessment Battery (NAB)^37^	A comprehensive set of subtests assessing cognitive domains like attention, memory, and executive function
**Psychiatric Questionnaires**	
Beck Depression Inventory (BDI)^38^	Assesses depression severity, including somatic and cognitive-affective symptoms
Hospital Anxiety and Depression Scale (HADS)^39^	Designed to measure anxiety and depression in patients with somatic conditions
Fatigue Severity Scale (FSS)^40^	Assesses fatigue severity, useful in conditions like hepatitis C and multiple sclerosis
**Neuroimaging Techniques**	
Magnetic Resonance Imaging (MRI)^41^	Assesses structural brain changes (atrophy, lesions, white matter abnormalities) to differentiate dementia types
Positron Emission Tomography (PET)^42^	Measures brain activity and glucose metabolism, aiding in diagnosing Alzheimer’s and neurodegenerative diseases
Functional MRI (fMRI)^43^	Assesses brain activity and connectivity, useful for studying cognitive processes in research
**Cognitive Impairment Assessment**	
Electronic Montreal Cognitive Assessment (eMoCA)^44^	Digital version of MoCA for portable cognitive assessment
Digital Clock Drawing Test (dCDT)^44^	Digital version of the Clock Drawing Test, used for assessing cognitive domains
Geras Solutions Cognitive Test (GSCT)^43^	A computerized test designed for screening and assessing cognitive functions
CogState^47^	A cognitive assessment using computerized tasks designed for detailed cognitive evaluation
Computerized Cognitive Screen (CoCoSc)^43^	Used for screening cognitive impairments and evaluating cognitive performance via computerized tasks
Virtual Reality-based Cognitive Tools^45^	Utilizes virtual reality and spatial navigation to assess cognitive function through interactive environments
**Artificial Intelligence-based Tool**	
Artificial Intelligence (AI) Algorithms^46^	Analyzes neuroimaging data and cognitive test results, enhancing diagnostic accuracy and early detection

When conducting neuropsychological tests, it is important to consider the influence of various sociodemographic factors. It has been established that age and education level are significant indicators that can have a considerable impact on cognitive test results. For instance, a recent study found that patients with chronic hepatitis C had the lowest neurocognitive scores and the highest levels of depressive symptoms compared to other groups examined, indicating a significant effect of HCV on patients.^47^

### Neuropsychological Tests

Neuropsychological tests are standardized assessments designed to measure specific cognitive functions. They help in differentiating between various types of cognitive impairments and determining baseline cognitive function. One of the most widely used tools, the Mini-Mental State Examination (MMSE), assesses various cognitive domains, including orientation, memory, attention, and language.^32^ It is commonly used for initial screening but may have limitations in detecting mild cognitive impairment or early stages of dementia. The Montreal Cognitive Assessment (MoCA) is designed to detect mild cognitive impairment and covers a broader range of cognitive domains compared to the MMSE, including executive function, visuospatial abilities, and attention.^13^,^33^ Evidence supports the use of a composite score, the MoCA-Clinical Frailty Score, developed from MoCA, to evaluate hepatic encephalopathy severity. The MoCA-Clinical Frailty Score composite score is effective in predicting deterioration in health-related and overall quality of life measures within six months. Recent data have also highlighted the prognostic value of a “multidimensional” frailty tool in predicting adverse clinical outcomes. This underscores the potential of a multifaceted approach to therapy, addressing cognitive impairment, physical frailty, and depression simultaneously.^34^ It is particularly useful for detecting subtle cognitive changes that may not be evident with other tests. Comprehensive batteries like the Wechsler Adult Intelligence Scale^35^ and the Cambridge Neuropsychological Test Automated Battery (CANTAB)^36^ provide detailed insights into various cognitive functions, including memory, executive function, and processing speed.^48^ Given the conflicting results on cognitive impairment in the literature, international researchers employ various comprehensive test batteries. One internationally recognized and widely used tool is the Wechsler Adult Reading Test, which enables assessment of baseline cognitive abilities before the onset of the disorder.^49^ Alternatively, the Neuropsychological Assessment Battery (NAB) can also be used, a well-validated, comprehensive set of subtests evaluating various cognitive domains such as attention, memory, and executive function. Each module within the NAB consists of several related subtests. Standardized scores for each subtest are derived based on demographically adjusted norms (age, gender, education), and standardized indices are calculated as the sum of the subtest performance scores within each module.^37^

### Psychiatric Questionnaires

The use of psychiatric questionnaires to detect cognitive impairment in patients with chronic viral hepatitis has been reported. The Beck Depression Inventory is a well-validated tool for assessing depression severity. It consists of 21 items that help identify two key factors of the disorder: the somatic factor, which includes symptoms such as loss of energy, changes in sleep patterns, irritability, changes in appetite, difficulty concentrating, fatigue, and loss of interest in sex; and the cognitive-affective factor, which includes symptoms such as sadness, pessimism, feelings of past failure, guilt, feelings of punishment, self-loathing, self-criticism, suicidal thoughts, crying, agitation, and worthlessness.^38^ Another effective method for diagnosing anxiety and depression is the Hospital Anxiety and Depression Scale, designed to measure mental stress in patients with somatic conditions.^39^

The Fatigue Severity Scale is a nine-item scale validated for use in patients with conditions such as hepatitis C, multiple sclerosis, and other chronic diseases. Despite the availability of numerous tools to assess fatigue, none provides both optimal specificity and sensitivity. This limitation contributes to the underestimation and the inadequate recognition and treatment of fatigue in patients.^40^ Given the absence of a single tool that encompasses all these components, it is crucial for researchers to identify which aspects of fatigue are most relevant to their study or patient population. They should then select a specific measure that best aligns with the relevant components of fatigue for their particular context.^44^

### Neuroimaging Techniques

Neuroimaging techniques are used to visualize brain structure and function, aiding in the diagnosis of cognitive impairments. Magnetic resonance imaging (MRI) is employed to assess structural brain changes such as atrophy, lesions, or white matter abnormalities. Structural MRI can help differentiate between conditions like Alzheimer’s disease and other types of dementia.^41^ Positron emission tomography (PET) imaging can be used to measure brain activity and glucose metabolism, providing information on functional changes and is useful in diagnosing Alzheimer’s disease and other neurodegenerative disorders by identifying characteristic patterns of brain metabolism.^42^ Functional MRI assesses brain activity by measuring changes in blood flow, providing insights into brain function and connectivity. It is used in research settings to explore cognitive processes and their neural underpinnings.^43^

### Cognitive Impairment Assessment

Recent research continues to advance the field of cognitive impairment diagnosis. New tools and techniques are being developed. Smartphone and tablet-based cognitive assessments offer portable and user-friendly options for monitoring cognitive function.^43^ The main cognitive assessment tools available are divided into three categories. Firstly, digital versions of traditional tests include electronic adaptations of conventional pen-and-paper tests. Examples are the electronic version of the Montreal Cognitive Assessment (eMoCA) and the digital Clock Drawing Test (dCDT). These traditional cognitive measures are adapted for computer administration and often focus on assessing specific cognitive domains.^44^ The second category includes new computerized neuropsychological products or test batteries specifically designed for screening, comprehensive assessment, or diagnostic purposes across multiple cognitive domains.^43^ Examples include: the Geras Solutions Cognitive Test (GSCT), CogState, Computerized Cognitive Screen (CoCoSc), Inoue, and CANTAB. The third category involves new data streams for cognitive assessment, specifically designed for computers or other mobile platforms and incorporating advanced technologies. This includes cognitive assessment tools that utilize virtual reality and spatial navigation technologies, often integrated into specialized games and interactive environments.^45^

### Artificial Intelligence

Artificial intelligence (AI) algorithms are increasingly used to analyze neuroimaging data and cognitive test results, potentially improving diagnostic accuracy and early detection.^46^ The AI can gain insights into cognition by studying the brain’s neural mechanisms, which are organized into networks within the cortex, based on uniform local structures, such as layers, columns, and basic local circuits. Furthermore, Surianarayanan et al. describe how collaboration between AI and neuroscience can enhance understanding of the brain’s mechanisms that generate human cognition. Artificial intelligence, supported by computational power, can facilitate large-scale simulations of neural processes, offering valuable insights into the generation of intelligence.^46^

### Determining the Appropriate Tool

The large number of tools available for assessing cognitive function reflects the complexity and variability of cognitive impairments, as well as the diverse needs of patients. Cognitive impairments can arise from a wide range of conditions, including neurodegenerative diseases, psychiatric disorders, and chronic illnesses, each with different manifestations and severity levels. As such, no single tool can effectively cover all possible clinical scenarios, which explains the proliferation of different instruments. These tools vary in their focus on specific cognitive domains, such as memory, attention, executive function, and language, as well as in their ability to detect subtle cognitive changes that might be missed by more general assessments. For instance, while the MMSE is commonly used for initial screening, it may fail to detect mild cognitive impairment or early stages of dementia. On the other hand, more comprehensive tools like the MoCA or Wechsler Adult Intelligence Scale offer a broader range of assessments, making them more suitable for detecting nuanced cognitive changes.

The variety of tools available underscores the importance of a personalized approach to cognitive assessment. Clinicians must consider the unique needs of each patient and select the most appropriate tool based on the cognitive domains they aim to assess, the stage of cognitive impairment, and the clinical context. For example, when evaluating a patient with suspected hepatic encephalopathy, the MoCA-Clinical Frailty Score (MoCA-CFS) composite score is particularly valuable, as it specifically assesses hepatic encephalopathy severity and predicts health-related outcomes. Meanwhile, for a broader cognitive assessment, neuropsychological batteries like the NAB or the CANTAB may be more appropriate. Additionally, with the increasing role of technology in healthcare, digital versions of traditional tests (e.g. eMoCA, dCDT) and new computer-based assessment tools (e.g. CogState, Geras Solutions Cognitive Test) offer convenient, portable, and user-friendly alternatives. These tools can be particularly useful for clinicians working in remote settings or where access to in-person assessments is limited. Finally, the integration of AI in neuroimaging and cognitive assessment offers new opportunities for improving diagnostic accuracy and early detection. Artificial intelligence can analyze large datasets from neuroimaging and cognitive test results, providing clinicians with more precise insights into brain function and enabling early intervention. This multifaceted approach ensures that clinicians can provide the most accurate diagnosis and treatment plan for their patient.

## IMPACT OF COGNITIVE IMPAIRMENT ON QUALITY OF LIFE

Patients with viral hepatitis experience a lower health-related quality of life compared to the general population. Factors contributing to this reduced quality of life in hepatitis C patients include: poor social adjustment, low perception of illness, high levels of stigmatization, impaired neurocognitive function, and high subjective physical symptoms.^48^,^49^ Additionally, some studies indicate that patients with chronic viral diseases, particularly hepatitis C, often report disproportionately high pain scores, which can further impair their functioning and quality of life. These factors are closely linked to symptoms of depression and underscore the importance of addressing both physical and mental health aspects in managing hepatitis C patients.^50^,^51^

Viral hepatitis C is known to diminish quality of life, which can be further exacerbated by various psychosocial factors and mental health conditions. The presence of pre-existing mental illnesses, such as depression, schizophrenia, and bipolar disorder, can complicate the management of HCV antiviral therapy. These mental health conditions may interfere with treatment adherence, response, and overall outcomes, highlighting the need for integrated care approaches that address both physical and mental health challenges in patients undergoing hepatitis C treatment.^50^

Poor quality of life and fatigue are also prevalent among patients with chronic viral hepatitis. Researchers suggest that there are significant links between demographic factors, psychological factors, and various quality of life indicators with fatigue. Addressing these factors through targeted interventions may help alleviate fatigue in these patients. By focusing on both the psychological and physical aspects of well-being, such interventions can potentially improve overall quality of life and reduce fatigue in individuals with chronic viral hepatitis.^52^–^54^ A recent comprehensive review revealed that, irrespective of liver disease severity, hepatitis C significantly reduces quality of life in most chronically infected patients. Surprisingly, there are relatively few studies assessing quality of life in patients with HBV infection. Various researchers have found that HCV-positive patients generally report significantly worse quality of life indicators compared to those with other liver diseases, with the exception of HBV infection. This is likely related to differences in the clinical course of the diseases, as chronic hepatitis B may progress in a less pronounced or less symptomatic form, whereas hepatitis C is often accompanied by more pronounced symptoms and higher risks of developing cirrhosis and liver cancer.^55^,^56^

Currently, a number of scales can be used to assess health-related quality of life in patients with viral hepatitis, allowing for an objective evaluation of various aspects of their physical condition. The three scales most commonly used in hepatitis patients are summarized in Table 2 and detailed below. The EuroQol 5 Dimensions 5 Levels (EQ-5D-5L) questionnaire was designed to assess health-related quality of life in patients with viral hepatitis. It provides a broad, general assessment of key health domains: mobility, self-care, daily activities, pain/discomfort, and anxiety/depression. The 5L version includes five additional levels of measurement for each parameter, providing a more precise grading of the patient’s condition across these domains.^57^ Another more detailed scale used to assess quality of life is the Short Form 36 Health Survey (SF-36). This questionnaire includes 36 questions that address eight dimensions of health for evaluating both physical and psychoemotional well-being. The questions are organized into two main categories: physical and psychoemotional health, encompassing various aspects of quality of life.^56^ Patients with chronic liver diseases often experience a variety of symptoms, and health-related quality of life (HRQOL) becomes a key aspect of their medical monitoring. The Chronic Liver Disease Questionnaire (CLDQ) was specifically developed to address the unique concerns of these patients. Comprising 29 questions, the CLDQ measures HRQOL and focuses on the impact of the disease on their physical, psychological, and social well-being of patients.^58^

**Table 2:** Summary of Scales for Assessing Quality of Life in Hepatitis Patients.

Scale	Key Measures	Key Features
EuroQol 5 Dimensions 5 levels (EQ-5D-5L)^57^	Mobility, self-care, daily activities, pain/discomfort, anxiety/depression	Utilizes five rating-scale questions aimed at obtaining a precise grading of health status, with five levels for each domain
Short Form 36 Health Survey (SF-36)^56^	Physical health, psychoemotional health	Asks 36 questions covering 8 health domains; useful for evaluating both physical and psychoemotional aspects
Chronic Liver Disease Questionnaire (CLDQ)^58^	Physical, psychological, and social well-being	Asks 39 questions specifically designed for chronic liver disease patients; used to asses various parameters related to quality of life

Based on this review, the most frequently used tools for assessing HRQOL in patients with viral hepatitis are the EQ-5D-5L, SF-36, and CLDQ. The EQ-5D-5L is commonly used because of its simplicity, precision, and more detailed granular assessment. Its brevity makes it a convenient tool for routine clinical use and large-scale studies. The SF-36 is frequently used because it offers a comprehensive evaluation of HRQOL; it is particularly beneficial in clinical trials and research settings due to its broad scope. The CLDQ, on the other hand, was specifically designed for chronic liver disease patients, making it a useful tool for specialized clinical practice in hepatology.

Evidence from this review and clinical practice indicates that there is no singular “preferred” tool for assessing HRQOL. Clinicians are advised to select the appropriate tool based on the context and objectives of their assessment. For general clinical practice and routine monitoring, the EQ-5D-5L is an excellent choice due to its simplicity and ease of administration. However, for more detailed assessments of psychoemotional aspects and comprehensive health evaluation, the SF-36 may be more suitable.

When choosing the right assessment tool, factors such as the specific health domains to be assessed, the patient population, the time required to administer the tool, and the desired level of detail should be considered. For specialized cases, like chronic liver diseases, using a tool designed specifically for that condition, such as CLDQ, may provide the most relevant insights.

## CONCLUSION

In summary, cognitive impairments in patients with viral hepatitis represent a critical area for further research and clinical attention. Addressing these impairments requires a multidisciplinary approach that includes effective management of the underlying viral infection, tailored cognitive assessments, and supportive interventions aimed at improving cognitive function and overall quality of life. Future research should focus on refining diagnostic tools, exploring the efficacy of cognitive rehabilitation strategies, and understanding the full spectrum of cognitive impacts associated with viral hepatitis. Enhanced awareness and targeted therapeutic strategies will be essential in mitigating the cognitive burden and improving patient outcomes.

## Abbreviations

AI: artificial intelligence
CANTAB: Cambridge Neuropsychological Test Automated Battery
CLDQ: Chronic Liver Disease Questionnaire
EQ-5D-5L: EuroQol 5 Dimensions 5 Levels
HBV: hepatitis B virus
HCV: hepatitis C virus
HRQOL: health-related quality of life
MoCA: Montreal Cognitive Assessment
MMSE: Mini-Mental State Examination
NAB: Neuropsychological Assessment Battery
SF-36: Short Form 36 Health Survey
